# Cinacalcet Rectifies Hypercalcemia in a Patient With Familial Hypocalciuric Hypercalcemia Type 2 (FHH2) Caused by a Germline Loss‐of‐Function Gα_11_ Mutation

**DOI:** 10.1002/jbmr.3241

**Published:** 2017-09-22

**Authors:** Caroline M Gorvin, Fadil M Hannan, Treena Cranston, Helena Valta, Outi Makitie, Camilla Schalin‐Jantti, Rajesh V Thakker

**Affiliations:** ^1^ Academic Endocrine Unit, Radcliffe Department of Medicine, Oxford Centre for Diabetes, Endocrinology and Metabolism (OCDEM) University of Oxford UK; ^2^ Department of Musculoskeletal Biology Institute of Ageing and Chronic Disease University of Liverpool UK; ^3^ Oxford Molecular Genetics Laboratory Churchill Hospital Oxford UK; ^4^ Children's Hospital University of Helsinki Helsinki Finland; ^5^ Folkhälsan Research Center Helsinki Finland; ^6^ Division of Endocrinology Abdominal Center University of Helsinki and Helsinki University Hospital Helsinki Finland

**Keywords:** PARATHYROID‐RELATED DISORDERS, DISORDERS OF CALCIUM/PHOSPHATE METABOLISM, THERAPEUTICS, CELL/TISSUE SIGNALING, ENDOCRINE PATHWAYS

## Abstract

G‐protein subunit α‐11 (Gα_11_) couples the calcium‐sensing receptor (CaSR) to phospholipase C (PLC)‐mediated intracellular calcium (Ca^2+^_i_) and mitogen‐activated protein kinase (MAPK) signaling, which in the parathyroid glands and kidneys regulates parathyroid hormone release and urinary calcium excretion, respectively. Heterozygous germline loss‐of‐function Gα_11_ mutations cause familial hypocalciuric hypercalcemia type 2 (FHH2), for which effective therapies are currently not available. Here, we report a novel heterozygous Gα_11_ germline mutation, Phe220Ser, which was associated with hypercalcemia in a family with FHH2. Homology modeling showed the wild‐type (WT) Phe220 nonpolar residue to form part of a cluster of hydrophobic residues within a highly conserved cleft region of Gα_11_, which binds to and activates PLC; and predicted that substitution of Phe220 with the mutant Ser220 polar hydrophilic residue would disrupt PLC‐mediated signaling. In vitro studies involving transient transfection of WT and mutant Gα_11_ proteins into HEK293 cells, which express the CaSR, showed the mutant Ser220 Gα_11_ protein to impair CaSR‐mediated Ca^2+^_i_ and extracellular signal‐regulated kinase 1/2 (ERK) MAPK signaling, consistent with diminished activation of PLC. Furthermore, engineered mutagenesis studies demonstrated that loss of hydrophobicity within the Gα_11_ cleft region also impaired signaling by PLC. The loss‐of‐function associated with the Ser220 Gα_11_ mutant was rectified by treatment of cells with cinacalcet, which is a CaSR‐positive allosteric modulator. Furthermore, in vivo administration of cinacalcet to the proband harboring the Phe220Ser Gα_11_ mutation, normalized serum ionized calcium concentrations. Thus, our studies, which report a novel Gα_11_ germline mutation (Phe220Ser) in a family with FHH2, reveal the importance of the Gα_11_ hydrophobic cleft region for CaSR‐mediated activation of PLC, and show that allosteric CaSR modulation can rectify the loss‐of‐function Phe220Ser mutation and ameliorate the hypercalcemia associated with FHH2. © 2017 The Authors. *Journal of Bone and Mineral Research* Published by Wiley Periodicals Inc.

## Introduction

G‐protein subunit alpha‐11 (Gα_11_) is a major downstream signaling partner of the cell‐surface calcium‐sensing receptor (CaSR), which is a family C G‐protein coupled receptor (GPCR) that plays a pivotal role in the parathyroid and renal regulation of extracellular calcium (Ca^2+^_e_) concentrations.^1^ Gα_11_ belongs to the G_q/11_ class of G‐proteins that enhance phospholipase C (PLC) activity, thereby leading to formation of: inositol 1,4,5‐trisphosphate (IP_3_), which induces rapid increases in intracellular Ca^2+^ (Ca^2+^_i_) concentrations; and enhances diacylglycerol (DAG) formation, which in turn activates protein kinase C and the mitogen‐activated protein kinase (MAPK) signaling cascade.^2^, ^3^ These signal transduction events allow the CaSR to respond to small fluctuations in the prevailing Ca^2+^_e_ concentrations ([Ca^2+^]_e_) and to induce alterations in parathyroid hormone (PTH) secretion and urinary calcium excretion.^4^

The identification of germline heterozygous mutations of Gα_11_, which is encoded by the *GNA11* gene on chromosome 19p13.3, that result in familial hypocalciuric hypercalcemia (FHH) has demonstrated the importance of this G‐protein subunit in Ca^2+^_e_ homeostasis.^5^ FHH is an autosomal dominant disorder characterized by lifelong elevations of serum calcium concentrations in association with normal or mildly raised serum PTH concentrations and low urinary calcium excretion (calcium‐to‐creatinine clearance ratio <0.01).^4^ FHH comprises three genetically distinct conditions, designated as FHH types 1 to 3. FHH1 (OMIM #145980) is caused by loss‐of‐function CaSR mutations.^1^ FHH2 (OMIM #145981) is caused by loss‐of‐function Gα_11_ mutations; to date, three FHH2‐associated mutations have been reported, comprising two missense mutations, Thr54Met and Leu135Gln, and an in‐frame isoleucine deletion at codon 200 (Ile200del)^5^, ^6^ (Supporting Fig.  1). FHH3 (OMIM #600740) is caused by loss‐of‐function mutations of the adaptor protein‐2 sigma subunit‐1 (*AP2S1*) gene, encoding AP2σ, which is involved in the clathrin‐mediated endocytosis of GPCRs, such as the CaSR.^1^

Positive allosteric modulators of the CaSR, known as calcimimetics, represent a targeted therapy for patients with symptomatic FHH. Indeed, cinacalcet, a licensed calcimimetic drug, has been used to ameliorate symptomatic hypercalcemia in patients with FHH1 and FHH3.^7^, ^8^, ^9^ However, such effects of cinacalcet in FHH2 patients have not been reported, although cinacalcet has been shown in vitro, to rectify signaling abnormalities associated with FHH2‐causing Gα_11_ mutations.^10^ Here, we report the effectiveness of cinacalcet in ameliorating the signaling defects and hypercalcemia due to a previously unreported FHH2‐associated Gα_11_ mutation, Phe220Ser.

## Patients and Methods

### Case report

**Figure 1:**
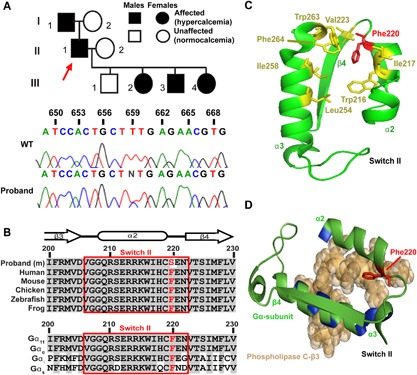
Identification and structural assessment of a Phe220Ser Gα_11_ mutation in a family with familial hypocalciuric hypercalcemia type 2. (*A*) The family comprised of 5 affected and 3 unaffected members (top). The proband (individual II.1) is indicated by an arrow. A heterozygous T‐to‐C transition at nucleotide c.659, predicted to result in a missense substitution of the WT Phe to a mutant (m) Ser at codon 220 of the Gα_11_ protein, was identified in the proband and confirmed to cosegregate with hypercalcemia by Sanger DNA sequence analysis. (*B*) Multiple protein sequence alignment of residues comprising the β3‐α2 loop, α2‐helix, and α2‐β4 loop that form switch II (red box) of Gα_11_ orthologs (top) and Gα‐subunit paralogs (bottom). Conserved residues are shown in gray, and WT (Phe, F) and mutant (Ser, S) residues are shown in red. (*C*) Structural model showing that Phe220 of Gα_11_ is part of a hydrophobic cluster of amino acids (yellow) within switch II and the α3‐helix, which stabilize the region in a conformation that facilitates effector binding. (*D*) Close‐up view of Phe220 residue, within the switch II region of the GTPase domain of Gα_11_, in complex with phospholipase C‐β3 (brown space‐filling model), with directly interacting residues of the α2 and α3 helices colored blue.

The proband (individual II.1, Fig. 1 *A*) was a 33‐year‐old male who presented with headaches, constipation, and pruritus. Biochemical investigations revealed hypercalcemia, hypophosphatemia, raised plasma PTH concentrations, and a low urine calcium‐to‐creatinine ratio (Table 1). A DXA scan showed normal bone mineral density values at the spine and hip (Table 1). A skin biopsy, undertaken to investigate the pruritus, demonstrated folliculitis (Table 1). His father, and three of his four children, also had hypercalcemia, constipation, and/or headaches (Table 1). Two of his affected children had eczema, and the proband's father had sustained an osteoporotic vertebral fracture. The familial hypercalcemia and reduced urinary calcium excretion were consistent with FHH; however, *CASR* or *AP2S1* mutations were not identified. Informed consent was obtained from individuals and where appropriate, parents and guardians of children, using protocols approved by the Research Ethics Committee of the Helsinki University Hospital.

**Table 1:** Clinical and Biochemical Findings in Available Affected Members of the Family With the Phe220Ser Gα_11_ Mutation

	Individuals			
Variable	II.1	III.2	III.3	III.4
Sex	Male	Female	Male	Female
Age at diagnosis	33 years	7 weeks	At birth	18 months
Associated clinical features	Headaches, pruritus, constipation^a^	Constipation, scoliosis, headaches^b^	Premature birth,^c^ eczema, constipation	Constipation, eczema
Serum biochemistry^d^				
Ionized calcium (mmol/L)	1.42	1.41	1.46	1.46
Phosphate (mmol/L)	0.50	1.98	1.81	1.62
Alkaline phosphatase (U/L)	77	275	461	243
Magnesium (mmol/L)	0.78	1.00	0.90	0.93
Creatinine (μmol/L)	76	26	78	26
PTH (ng/L)	98	39	100	40
25‐hydroxyvitamin D (nmol/L)	49	50	102	122
Thyrotropin (TSH) (mU/L)	2.82	1.83	5.5^e^	1.53
Urinary calcium excretion	0.09^f^	0.002	0.001	0.003

^a^ A skin biopsy demonstrated folliculitis with keratin within the widened hair follicle and inflammatory cells around the hair follicle. Normal bone mineral density *T*‐score ≥ –1.0 at lumbar spine and femoral neck.

^b^ Individual III.2 (Fig. 1) developed headaches from age 10 years.

^c^ Individual III.3 was born prematurely at gestational age 27+3 weeks.

^d^ Normal biochemical ranges: ionized calcium, 1.16 to 1.30 mmol/L (adults) and 1.16 to 1.39 mmol/L (age 1 to 12 months); phosphate, 1.50 to 2.60 mmol/L (neonates), 1.50 to 2.50 mmol/L (age 1 to 6 months), 1.20 to 1.80 mmol/L (age 2 to 12 years), and 0.71 to 1.53 mmol/L (adults); alkaline phosphatase activity, 35 to 105 U/L (adults) and 115 to 460 U/L (age 15 days to 1 year); magnesium, 0.71 to 0.94 mmol/L (adults) and 0.7 to 1.0 mmol/L (age 3 months to 17 years); creatinine, 60 to 100 μmol/L (adult males) and 10 to 56 μmol/L (age 8 days to 2 years); PTH, 8 to 73 ng/L; 25 hydroxyvitamin D, >50 nmol/L; thyrotropin; 0.5 to 3.6 mU/L (age >14 weeks) and 0.4 to 7 mU/L (age 11 to 14 weeks); calcium‐to‐creatinine clearance ratio >0.02.

^e^ TSH concentration measured in umbilical cord blood (normal range not available for premature infant cord blood TSH).

^f^ Calcium‐to creatinine ratio, normal 0.3‐0.7.

### Mutational analysis

DNA sequence analyses of *GNA11* exons 1 to 7 and adjacent splice sites (NM_002067) was performed using leukocyte DNA and gene‐specific primers (Sigma‐Aldrich, Gillingham, UK), as reported.^5^, ^6^ Polymorphic variants were identified from public databases (Supporting Table  1).

### Protein sequence alignment and three‐dimensional modeling of Gα_11_ structure

Protein sequences of Gα_11_ orthologs were aligned using ClustalOmega (European Bioinformatics Institute [EMBL‐EBI], Cambridgeshire, UK; http://www.ebi.ac.uk/Tools/msa/clustalo/).^6^ Gα_11_ three‐dimensional modeling was undertaken using the reported three‐dimensional structures of: Gα_q_ in complex with phospholipase C‐β3 (Protein Data Bank accession no. 3OHM)^11^; Gα_q_ in complex with the small molecule inhibitor YM‐254890 (Protein Data Bank accession no. 3AH8)^12^; and Gα_q_ in complex with the regulator of G‐protein signaling 2 (RGS2) (Protein Data Bank accession no. 4EKC and 4QJ3).^13^, ^14^ The Gα_q_ protein, which shares 90% identity at the amino acid level with Gα_11_, was used because crystal structures of Gα_11_ are not available. Figures were prepared using the PyMOL Molecular Graphics System, Schrodinger.

### Cell culture and transfection

Mutations were introduced into the previously described pBI‐CMV2‐*GNA11* expression construct by site‐directed mutagenesis using the Quikchange Lightning Kit (Agilent Technologies, Santa Clara, CA, USA) and gene‐specific primers (Sigma‐Aldrich). Wild‐type (WT) or mutant pBI‐CMV2‐*GNA11* constructs were transiently transfected into HEK293 cells stably expressing CaSR (HEK‐CaSR) using Lipofectamine 2000 (Life TechnologiesCarlsbad, CA, USA), as described.^15^, ^16^ The pBI‐CMV2‐*GNA11* bidirectional vector allows for co‐expression of Gα_11_ and GFP at equivalent levels.^5^ HEK‐CaSR cells were maintained in DMEM‐Glutamax media (Gibco, Carlsbad, CA, USA) that has a calcium concentration of 1.80mM. The presence of mutations was verified using dideoxynucleotide sequencing with the BigDye Terminator v3.1 Cycle Sequencing Kit (Life Technologies) and an automated detection system (ABI3730 Automated capillary sequencer; Applied Biosystems, Carlsbad, CA, USA).^17^ Luciferase reporter constructs (pGL4.30‐NFAT, pGL4.33‐SRE) were purchased from Promega. HEK293 cells were used because suitable parathyroid and renal tubular cells are not available, and HEK293 cells have been established as a model for the functional expression of Gα_11_ proteins.^5^, ^15^, ^16^ HEK‐CaSR cells were cultured in high‐glucose DMEM (Invitrogen, Carlsbad, CA, USA) supplemented with 10% fetal bovine serum and 1% Geneticin at 37°C, 5% CO_2_.^5^ Successful transfection was confirmed by visualizing GFP fluorescence using an Eclipse E400 fluorescence microscope with an epifluorescence filter, and images were captured using a DXM1200C digital camera and NIS Elements software (Nikon, Kingston‐Upon‐Thames, UK).^5^, ^16^ The expression of Gα_11_ and CaSR proteins was confirmed by Western blot analyses using Gα_11_ (Santa Cruz Biotechnologies), anti‐calnexin (Millipore, Billerica, MA), or anti‐CaSR (Abcam, Cambridge, UK) antibodies. Calnexin, a housekeeping protein, was used as a loading control. The Western blots were visualized using an Immuno‐Star Western C kit (BioRad, Hercules, CA, USA) on a BioRad Chemidoc XRS+ system.^6^

### Fluo‐4 intracellular calcium assay

Ca^2+^_e_‐induced Ca^2+^_i_ responses were measured by Fluo‐4 calcium assays adapted from published methods.^18^ HEK‐CaSR cells were plated in poly‐L‐lysine treated black‐walled 96‐well plates (Corning, Corning, NY, USA), and transiently transfected with 1000 ng/mL pBI‐CMV2‐*GNA11*. Cells were conditioned in serum‐free media (1.8mM Ca^2+^_e_) overnight, then incubated in Ca^2+^‐free and Mg^2+^‐free Hanks Balanced Salt Solution (HBSS) for 1 hour, followed by loading with Fluo‐4 dye, prepared according to manufacturer's instructions (Invitrogen). Cells were loaded for 40 min at 37°C, then either a 20% aqueous solution of 2‐hydoxypropyl‐β‐cyclodextrin (vehicle), or 30nM or 100nM cinacalcet was added. Cells were then incubated for a further 20 min at 37°C.^18^ Baseline measurements were made and CaCl_2_ was injected into each well to increase the [Ca^2+^]_e_ in a stepwise manner from 0.5 to 10mM [Ca^2+^]_e_, using an automated system. Changes in Ca^2+^_i_ were recorded on a PHERAstar instrument (BMG Labtech, Aylesbury, UK) at 37°C with an excitation filter of 485 nm and an emission filter of 520 nm. The peak mean fluorescence ratio of the transient response after each individual stimulus was measured using MARS data analysis software (BMG Labtech), and expressed as a normalized response. Nonlinear regression of concentration‐response curves was performed with GraphPad Prism (GraphPad Software, Inc., La Jolla, CA, USA) using the normalized response at each [Ca^2+^]_e_ for each separate experiment for the determination of the EC_50_ (ie, [Ca^2+^]_e_ required for 50% of the maximal response). Assays were performed in four to 12 biological replicates for each of the expression constructs. Statistical analysis was performed using the *F*‐test.^5^, ^6^

### Luciferase reporter assay

HEK‐CaSR cells were seeded in 48‐well plates and transiently transfected with 100 ng/mL pBI‐CMV2‐*GNA11* WT or mutant proteins, 100 ng/mL luciferase construct (either pGL4‐NFAT or pGL4‐SRE) and 10 ng/mL pRL control vector for 48 hours. Cells were incubated in serum‐free media for 12 hours, followed by treatment of cells for 4 hours with 0.1mM to 10mM CaCl_2_. Cells were lysed and assays performed with Dual‐Glo luciferase (Promega, Madison, WI, USA) on a Veritas Luminometer (Promega), as described.^19^ Luciferase:renilla ratios were expressed as fold‐changes relative to responses at basal CaCl_2_ concentrations. For studies with cinacalcet (Cambridge Bioscience, Cambridge, UK), the drug was added to cells, as described.^10^, ^19^ All assays were performed using four biological replicates on up to three independent occasions. Statistical analysis was performed by two‐way ANOVA with Tukey's multiple‐comparisons test using GraphPad Prism 6.

### Measurement of ERK phosphorylation

HEK‐CaSR cells were seeded in 48‐well plates and transfected with 200 ng/mL WT or mutant Gα_11_, 48 hours prior to performance of assays. Transfected cells were incubated in serum‐free media 12 hours prior to treatment of cells with 0.1mM to 10mM CaCl_2_. Cells were then lysed in Surefire lysis buffer, and AlphaScreen Surefire ERK assays (PerkinElmer, Beaconsfield, UK) measuring phosphorylated and total proteins were performed, as described.^10^, ^19^ The fluorescence signal in both assays was measured using the PHERAstar FS microplate reader (BMG Labtech).^19^ The ratio of phosphorylated ERK (pERK) to total ERK measured at each [Ca^2+^]_e_ were normalized to the mean responses of WT‐expressing cells and expressed as a fold‐change of responses obtained at basal (0.1mM) [Ca^2+^]_e_. All assay conditions were performed in four biological replicates on up to four independent occasions. Statistical analysis was performed by two‐way ANOVA with Tukey's multiple‐comparisons test using GraphPad Prism 6.

### Web resources

Predicted effect of the mutation was assessed using Polyphen‐2 (http://genetics.bwh.harvard.edu/pph2/)^20^ and MutationTasting (http://www.mutationtaster.org/).^21^

### Statistics

All studies involved between four and 16 separate transfection experiments. Statistical analyses for Ca^2+^_i_ EC_50_ used the *F‐*test,^19^ and two‐way ANOVA with Tukey's multiple‐comparisons test was used for pERK and luciferase reporter assays. Analyses were undertaken using GraphPad Prism, and a value of *p* < 0.05 was considered significant.

## Results

### Identification of a novel Phe220Ser Gα_11_ mutation in a family with FHH2

DNA sequence analysis of *GNA11* in the proband identified a heterozygous T‐to‐C transition at nucleotide c.659 (Fig. 1 *A*), resulting in a missense substitution of the WT Phe to a mutant Ser at residue 220 of the Gα_11_ protein. Bioinformatic analyses predicted the Phe220Ser variant to be damaging and likely disease‐causing (Polyphen‐2 score = 1, MutationTasting score = 0.99). The absence of this DNA sequence abnormality in >67,200 exomes from the NHLBI‐ESP and Exome Aggregation Consortium cohorts, together with evolutionary conservation of the Phe220 residue in Gα_11_ orthologs and Gα‐subunit paralogs (Fig. 1 *B*), also indicated that the Phe220Ser abnormality likely represents a pathogenic mutation rather than a benign polymorphic variant.

### The Phe220Ser mutation is located in the switch II region of Gα_11_ and predicted to impair PLC activation

Homology modeling, using crystal structures of the related Gα_q_ protein,^11^, ^12^, ^22^ revealed the WT Phe220 residue to be located within the α2‐helix of the flexible switch II region of the Gα_11_ GTPase domain (Fig. 1 *B*, *C*; Supporting Fig. 1), and to comprise part of a hydrophobic cluster of amino acids located within a cleft region formed by switch II and the adjacent α3‐helix that interact with PLC upon G‐protein activation (Fig. 1 *C*, *D*).^11^ Substitution of the nonpolar hydrophobic WT Phe220 residue, with the mutant polar hydrophilic Ser220 residue is predicted to disrupt Gα_11_ function by impairing PLC activation and signaling.

### The Gα_11_ Phe220Ser mutation impairs Ca^2+^_i_ responses

**Figure 2:**
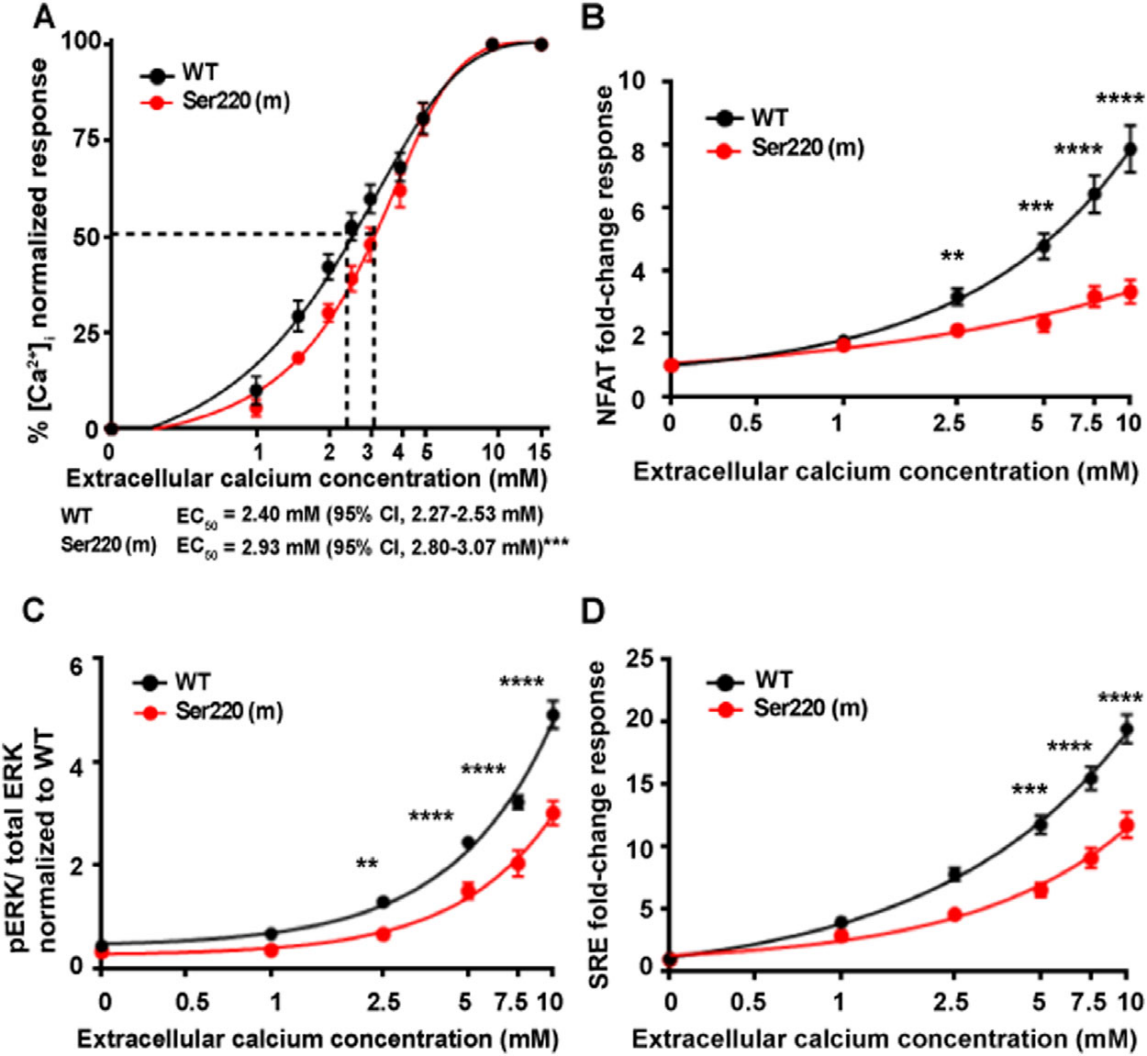
Effect of the mutant Ser220 Gα_11_ on Ca^2+^_i_ and ERK signaling in HEK‐CaSR cells. (*A*) Ca^2+^_i_ responses to changes in [Ca^2+^]_e_ of HEK‐CaSR cells expressing WT (Phe220) or mutant (Ser220) Gα_11_ proteins shown as the mean ± SE of 4 to 11 transfections. The Ser220 Gα_11_ mutant caused a rightward shift in the Ca^2+^_i_ concentration‐response curve with significantly increased EC_50_ value compared to WT cells (***p < 0.0001, *F*‐test). (*B*) [Ca^2+^]_e_‐induced NFAT reporter responses, which are activated by elevations of Ca^2+^_i_, were assessed in HEK‐CaSR cells expressing WT or mutant Gα_11_ proteins. Responses at each [Ca^2+^]_e_ are expressed as a fold‐change of basal [Ca^2+^]_e_ responses, and shown as mean ± SE of 12 transfections. NFAT luciferase reporter activity increased in all cells in a concentration‐dependent manner, and was significantly reduced in Ser220 cells compared to WT cells. (*C*) Ca^2+^_e_‐induced pERK responses of cells expressing WT or mutant Gα_11_ protein, expressed as a ratio to total ERK levels, and shown as mean ± SE of 8 transfections. (*D*) Ca^2+^_e_‐induced SRE reporter responses of cells expressing WT or mutant Gα_11_ protein, expressed compared to basal SRE reporter activity, and shown as mean ± SE of 8 transfections. The Ser220 Gα_11_ mutant was associated with significantly decreased pERK and SRE reporter responses compared to WT. **p < 0.01 and ****p < 0.0001, by a 2‐way ANOVA with Tukey's multiple‐comparisons test.

The Phe220Ser mutation is predicted to diminish PLC activation, and we therefore assessed its effects on Ca^2+^_i_ mobilization, which has previously been shown to be enhanced downstream of PLC by the G_q/11_ proteins.^23^, ^24^, ^25^, ^26^ HEK‐CaSR cells were transiently transfected with pBI‐CMV2‐*GNA11* constructs expressing the WT (Phe220) or mutant (Ser220) Gα_11_ proteins (Supporting Fig.  2). The Ca^2+^_i_ responses of the mutant Ser220 Gα_11_‐expressing HEK‐CaSR cells, revealed that a significantly greater [Ca^2+^]_e_ was required to achieve half‐maximal (EC_50_) Ca^2+^_i_ responses, when compared with WT‐expressing cells (Fig. 2 *A*). Thus, the Ser220 mutant‐expressing cells showed a rightward shift in the concentration‐response curve, with a significantly elevated mean EC_50_ value (*p* < 0.001) of 2.93mM (95% confidence interval [CI], 2.80 to 3.07mM), compared with 2.40mM (95% CI, 2.27 to 2.53mM) for WT‐expressing cells. The Ca^2+^_i_ responses were also assessed by measurement of nuclear factor of activated T‐cells (NFAT), which is a downstream mediator of Ca^2+^_i_ signaling.^27^ NFAT fold‐change responses were determined using a luciferase reporter assay, and these studies showed that HEK‐CaSR cells expressing the Ser220 Gα_11_ mutant had a significantly reduced fold‐change response (fold‐change = 2.3 ± 0.3) following exposure to 5mM [Ca^2+^]_e_, when compared with WT cells (fold‐change = 4.8 ± 0.4, *p* < 0.001) (Fig. 2 *B*). These data demonstrated that the Gα_11_ Phe220Ser mutation impairs CaSR‐mediated Ca^2+^_i_ responses, consistent with a loss‐of‐function associated with FHH2.

### The Gα_11_ Phe220Ser mutation impairs ERK phosphorylation

The ERK protein represents a major component of the MAPK signaling cascade, which has previously been shown to be activated by PLC.^3^, ^28^ We assessed the effect of the Gα_11_ Phe220Ser mutation on ERK phosphorylation by measuring changes in pERK in response to increases in Ca^2+^_e_ in WT and mutant Ser220 Gα_11_‐expressing cells using the AlphaScreen assay, and also by assessing serum‐response element (SRE)‐mediated transcriptional activity, which is regulated by ERK signaling.^19^ The pERK responses of the Ser220 Gα_11_ mutant were shown to be significantly reduced. Thus, the Ser220 Gα_11_ mutant led to significant reductions in: pERK‐to‐total ERK ratios (Ser220 response at 5mM Ca^2+^_e_ = 3.7 ± 0.4 versus Phe220 = 6.1 ± 0.3, *p* < 0.0001) (Fig. 2 *C*); and to SRE reporter fold‐changes (Ser220 fold‐change at 5mM Ca^2+^_e_ = 6.5 ± 0.5 versus Phe220 fold‐change = 11.8 ± 0.7, *p* < 0.0001) (Fig. 2 *D*). Thus, the Gα_11_ Phe220Ser mutation impaired CaSR‐mediated ERK phosphorylation.

### Phe220 forms part of a hydrophobic cluster of Gα_11_ residues required for PLC‐mediated signaling

**Figure 3:**
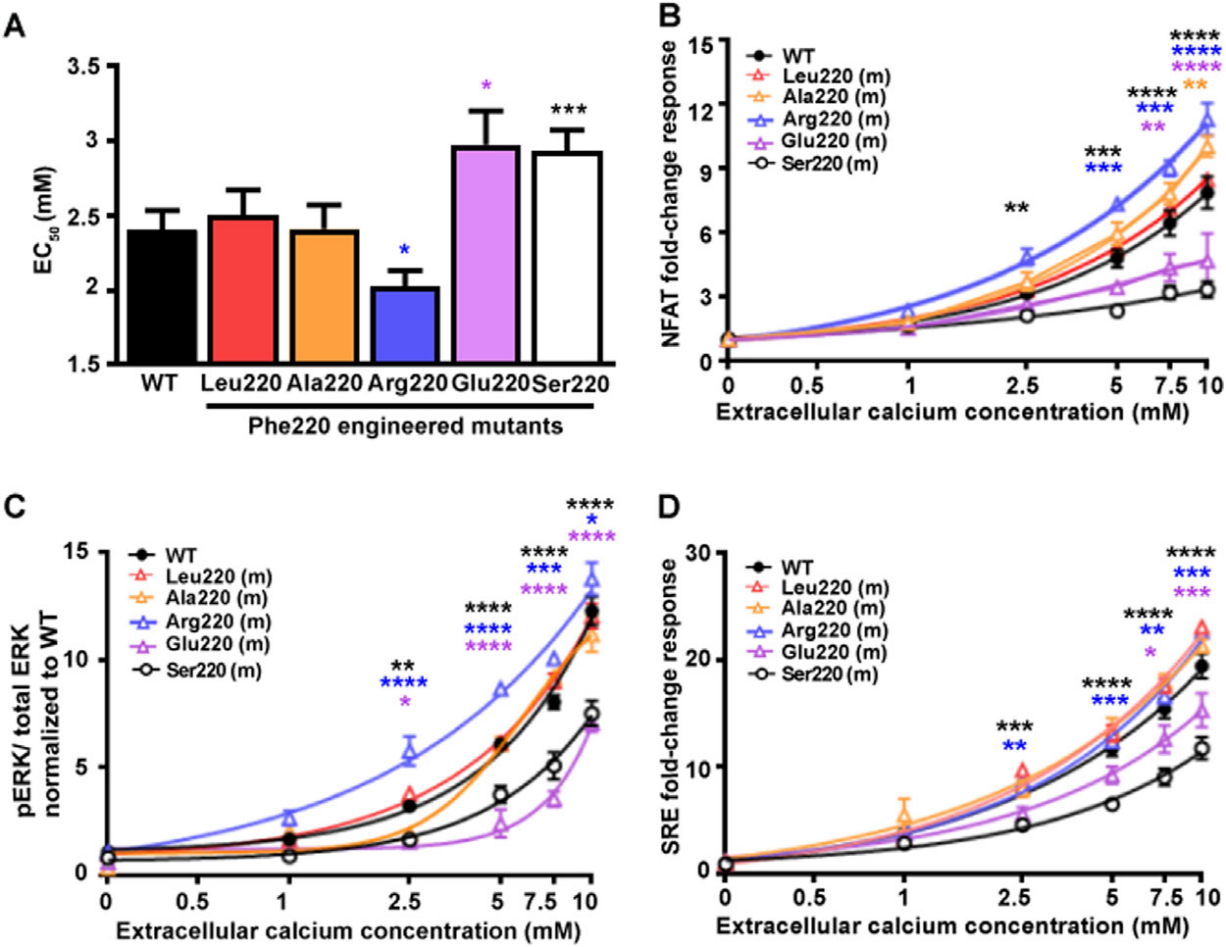
Effect of Gα_11_ residue 220 hydrophobicity on PLC‐mediated signaling. (*A*) Histograms showing Ca^2+^_e_‐induced Ca^2+^_i_ EC_50_ values with 95% confidence intervals (CIs) for cells expressing WT Phe220 or residue 220 mutant (m) Gα_11_ proteins from 6 to 12 transfections. Statistical analyses were performed using the *F‐*test. The Arg220 Gα_11_ mutant reduced the EC_50_ value, whereas the Glu220 and Ser220 Gα_11_ mutants elevated the EC_50_ value. (*B*) Ca^2+^_e_‐induced NFAT reporter responses, which assess Ca^2+^_i_ signaling, of cells expressing WT or residue 220 mutant Gα_11_ proteins. Responses at each [Ca^2+^]_e_ are expressed as a fold‐change of basal [Ca^2+^]_e_ responses, and shown as mean ± SE of 4 to 12 transfections. NFAT luciferase reporter activity increased in all cells in a concentration‐dependent manner, however responses were significantly elevated in Arg220 Gα_11_ mutant, and reduced in Glu220 Gα_11_ mutant cells, compared to WT cells. (*C*) Ca^2+^_e_‐induced pERK responses of cells expressing WT or mutant residue 220 Gα_11_ proteins, expressed as a ratio to total ERK levels. The Arg220 mutant was associated with significantly increased responses, and the Glu220 and Ser220 mutants were associated with decreased responses, when compared to WT. Data are shown as mean ± SE of 4 to 8 independent transfections. (*D*) Ca^2+^_e_‐induced SRE reporter responses of cells expressing WT or mutant (m) residue 220 Gα_11_ proteins, expressed compared to basal SRE reporter activity. The engineered Arg220 mutant was associated with significantly increased, and the engineered Glu220 and FHH2‐associated Ser220 mutants resulted in decreased responses, when compared to WT. Data are shown as mean ± SE of 4 to 8 independent transfections. Statistical analyses performed using 2‐way ANOVA with Tukey's multiple‐comparisons test comparing WT with Gα_11_ mutants Ala220, Arg220, Glu220, and Ser220. *p < 0.05, **p < 0.01, ***p < 0.001, ****p < 0.0001.

**Figure 4:**
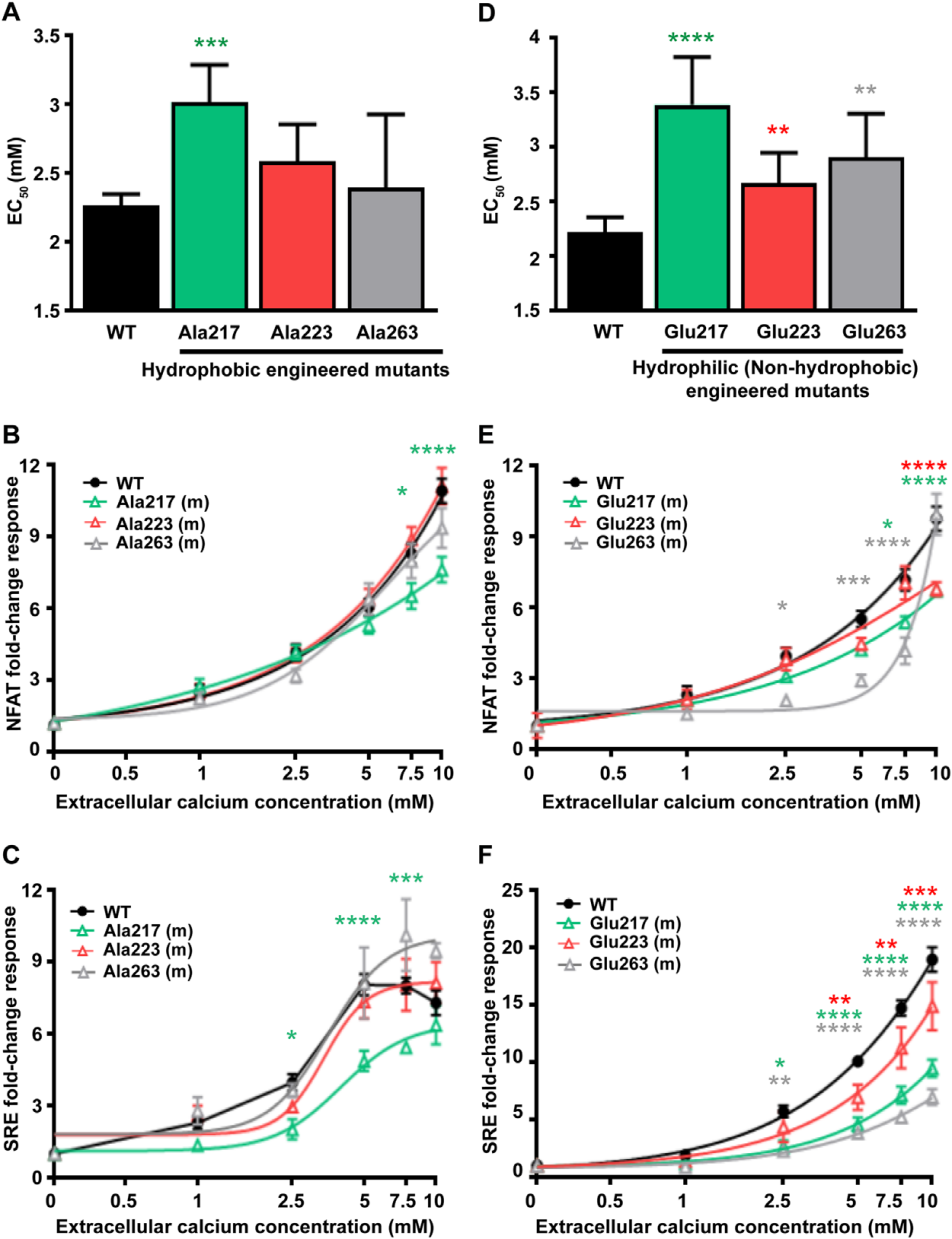
Effect of mutating residues within the Gα_11_ switch II‐α3 hydrophobic cluster on PLC‐mediated signaling. (*A*) Histograms showing Ca^2+^_e_‐induced Ca^2+^_i_ EC_50_ values with 95% CI for cells expressing WT and alanine (Ala) Gα_11_ mutants (m) of residues 217, 223, and 263 Gα_11_ proteins from 4 to 8 independent transfections. Only the Ala217 Gα_11_ mutant increased the EC_50_ value, when compared to WT cells. Statistical analyses were performed using the *F‐*test. (*B*, *C*) Ca^2+^_e_‐induced (*B*) NFAT and (*C*) SRE luciferase reporter responses of cells expressing WT or alanine (Ala) Gα_11_ mutants. Responses at each [Ca^2+^]_e_ are expressed as a fold‐change of basal [Ca^2+^]_e_ responses, and shown as mean ± SE of 4 to 8 transfections. NFAT and SRE reporter responses were significantly reduced in Ala217‐expressing cells compared to WT cells. (*D*) Histograms showing Ca^2+^_e_‐induced Ca^2+^_i_ EC_50_ values with 95% CI for cells expressing WT and glutamic acid (Glu) Gα_11_ mutants (m) of residues 217, 223, and 263 from 4 to 8 independent transfections. All Glu Gα_11_ mutants increased the EC_50_ value, when compared to WT cells. Statistical analyses were performed using the *F‐*test. (*E*, *F*) Ca^2+^_e_‐induced (*E*) NFAT and (*F*) SRE luciferase reporter responses of cells expressing WT or glutamic acid (Glu) Gα_11_ mutants. Responses at each [Ca^2+^]_e_ are expressed as a fold‐change of basal [Ca^2+^]_e_ responses, and shown as mean ± SE of 4 to 8 transfections. NFAT and SRE reporter responses were significantly reduced in cells expressing all 3 glutamic acid mutants compared to WT cells. Statistical analyses for *B*, *C*, *E*, and *F* were performed using 2‐way ANOVA with Tukey's multiple‐comparisons test comparing WT to mutant 217 (green), 223 (red), and 263 (gray) Gα_11_ proteins. *p < 0.05, ***p < 0.001, ****p < 0.0001.

To further determine the mechanistic role of the Phe220 residue and the importance of residue 220 hydrophobicity for Gα_11_ function and PLC‐mediated signaling (Fig. 1 *C*), we engineered mutations of Phe220 to Leu and Ala, which are hydrophobic residues, and to Arg and Glu, which are hydrophilic positively‐charged and negatively‐charged residues, respectively, and studied their effect on Ca^2+^_i_ and ERK responses following transient expression in HEK‐CaSR cells (Fig. 3). The Leu220 and Ala220 Gα_11_ mutants did not alter Ca^2+^_i_ responses (Fig. 3 *A*, *B*; Supporting Fig. 3), or ERK activity, as measured using the pERK AlphaScreen and SRE reporter assays (Fig. 3 *C*, *D*). In contrast, the positively‐charged Arg220, and negatively‐charged Glu220 Gα_11_ mutants, respectively, enhanced and impaired Ca^2+^_i_ and ERK signaling responses in HEK‐CaSR cells (Fig. 3, Supporting Fig.  3). Thus, these findings demonstrated that a hydrophobic residue at position 220 is required for signaling via PLC. The enhancement of CaSR‐mediated signaling by the Arg220 Gα_11_ mutant may be explained by its structural effect on the G_11_ heterotrimer. Homology modeling, using crystal structures of the related G_q_ protein in complex with Gβγ,^12^ revealed that the Arg220 residue faces away from the hydrophobic cluster, into the region at which Gα and Gβγ bind, a hydrophobic region that is necessary for Gα activation^29^ (Supporting Fig.  4). Disruption of this hydrophobic region has been shown to cause constitutive activation,^29^ and modeling predicts that the Arg220 residue, which is charged and hydrophilic, disrupts this region, thus enhancing activation by impairing Gαβγ association (Supporting Fig.  4). To investigate the importance of the Gα_11_ hydrophobic cleft, formed by switch II and the α3‐helix (Fig. 1 *C*, *D*), for PLC activation, we engineered mutations of three other hydrophobic residues, Ile217, Val223, and Trp263, within this region, to either an Ala residue, to retain hydrophobicity, or to a Glu residue, to lose hydrophobicity, and studied the effects of these mutants on Ca^2+^_i_ and ERK responses following their transient expression in HEK‐CaSR cells (Supporting Fig.  5). This demonstrated that substitution to Ala of only one hydrophobic residue, Ile217, altered PLC‐mediated signaling (Fig. 4 *A*–*C*; Supporting Fig.  6). In contrast, substitution of any of the Ile217, Val223, and Trp263 hydrophobic residues to a Glu residue led to a significant reduction in Ca^2+^_i_ and ERK responses (Fig. 4 *D*–*F*, Supporting Fig.  6). Thus, hydrophobicity of the switch II‐α3 cleft is important for PLC activation.

### Cinacalcet rectifies the impaired PLC signaling responses and hypercalcemia caused by the Phe220Ser Gα_11_ mutation

**Figure 5:**
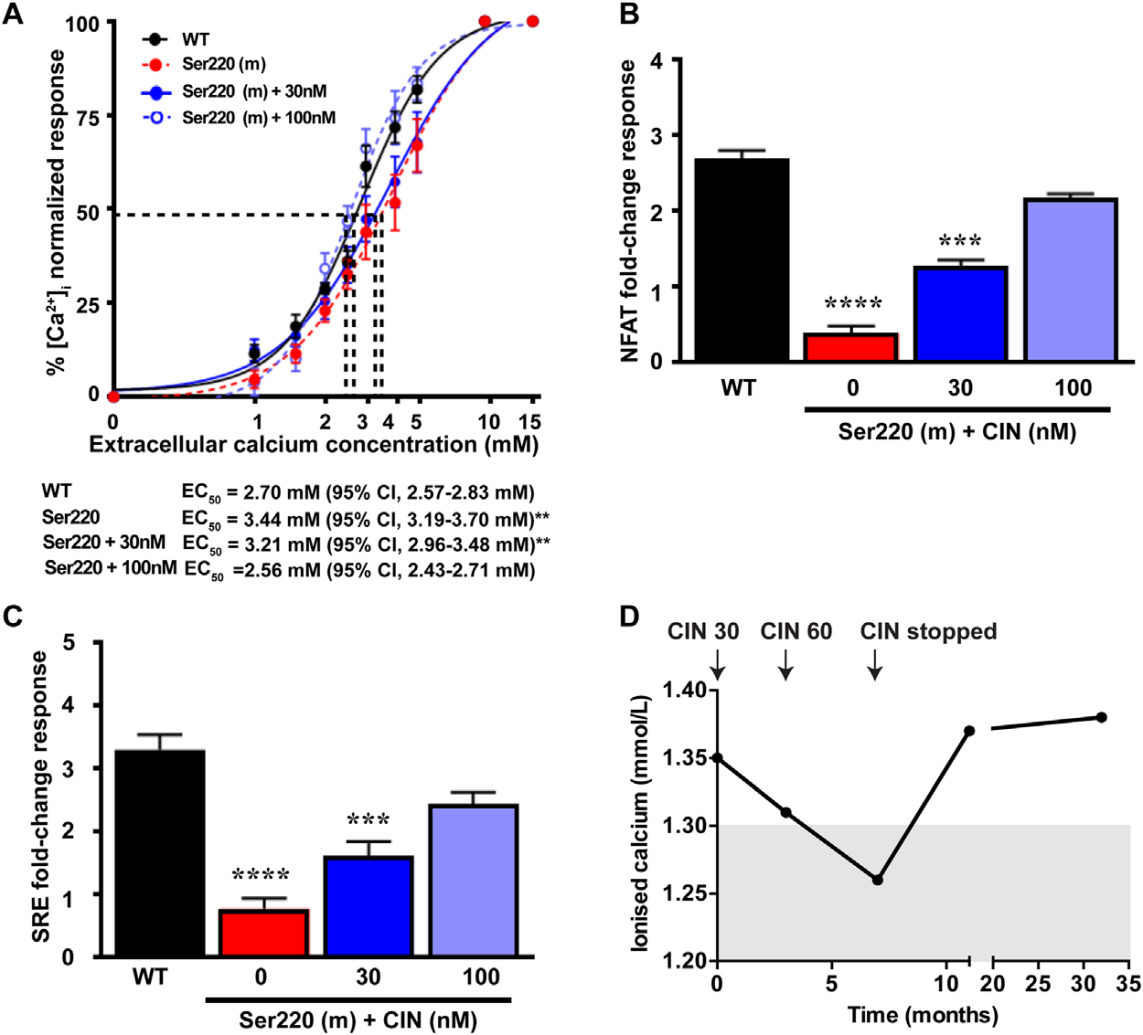
Effect of cinacalcet on the loss‐of‐function and hypercalcemia caused by the Phe220Ser Gα_11_ mutation. (*A*) Effect of cinacalcet on Ca^2+^_i_ responses to changes in [Ca^2+^]_e_ of HEK‐CaSR cells transfected with WT or Ser220 Gα_11_ mutant (m). The Ca^2+^_i_ responses to changes in [Ca^2+^]_e_ are expressed as a percentage of the maximum normalized responses and shown as the mean ± SE of 6 to 12 independent transfections. The Ser220 Gα_11_ mutant led to a rightward shift in the concentration‐response curve. The addition of 30nM cinacalcet, a concentration previously shown to rectify the loss‐of‐function associated with the reported Leu135Gln Gα_11_ FHH2 mutant in vitro,^10^ had no effect on Ser220 responses. However, addition of 100nM cinacalcet rectified the rightward shift of the Ser220 Gα_11_ mutant, such that it was not different to WT responses. (*B*, *C*) Histograms showing (*B*) NFAT and (*C*) SRE luciferase reporter activity in response to 5mM Ca^2+^_e_ in HEK‐CaSR cells expressing WT or Ser220 mutant constructs, treated with vehicle or cinacalcet (CIN). Data are shown as mean ± SE of 4 independent transfections; 100nM cinacalcet was required to significantly increase NFAT and SRE reporter activity to WT levels. The responses of cells expressing WT or mutant Gα_11_ proteins were compared using the *F*‐test for *A* and 2‐way ANOVA with Tukey's multiple‐comparisons test for *B* and *C*. **p < 0.01, ***p < 0.001, ****p < 0.0001. (*D*) Effect of cinacalcet on the serum ionized calcium concentrations of the FHH2 proband (individual II.1, Fig. 1 *A*) with the Phe220Ser Gα_11_ mutation. Arrows indicate initiation of oral cinacalcet at 30 mg/day (CIN 30), at 60 mg/day (CIN 60), and its discontinuation. Cinacalcet treatment decreased serum concentrations of ionized calcium and PTH from 98 ng/L (pretreatment) to 58 ng/L (posttreatment) (normal range, 8 to 73 ng/L). Shaded area indicates normal range.

To determine whether cinacalcet may be an effective therapy for FHH2, we evaluated the effects of different cinacalcet concentrations (0, 30, and 100nM) on the altered PLC signaling responses due to the Phe220Ser Gα_11_ mutant. Treatment of mutant Ser220 Gα_11_‐expressing HEK‐CaSR cells with 30nM cinacalcet, failed to normalize the impaired Ca^2+^_i_ and SRE reporter responses (Fig. 5 *A*–*C*); however, 100nM cinacalcet, reduced the EC_50_ values, and increased NFAT and SRE reporter activity (Fig. 5 *A*–*C*), such that these values were not significantly different from untreated WT cells. These in vitro findings suggested that higher doses of cinacalcet may be required for the treatment of patients harboring the Phe220Ser Gα_11_ mutation. Cinacalcet was administered to the FHH2 proband as he had symptoms such as headaches, constipation, and pruritus (Table 1), which may have been caused by the hypercalcemia. The proband was initially commenced on 30 mg/day of oral cinacalcet for 3 months, and in keeping with the above cellular studies, which showed a lack of efficacy when using low cinacalcet concentrations, the 30 mg/day dose failed to normalize his elevated serum calcium concentrations (Fig. 5 *D*). However, increasing the dose of cinacalcet to 60 mg/day, successfully lowered the serum concentrations of: ionized calcium from 1.42 to 1.26 mmol/L (normal range, 1.16 to 1.30 mmol/L) (Fig. 5 *D*); and PTH from 98 to 58 ng/L (normal range, 8 to 73 ng/L). Although the proband became normocalcemic on cinacalcet, the pruritus, from which he suffered the most, did not resolve, and cinacalcet 60 mg/day was discontinued after 4 months. His hypercalcemia returned upon cessation of cinacalcet therapy (Fig. 5 *D*).

## Discussion

Our studies have identified a previously unreported FHH2‐causing heterozygous Gα_11_ germline loss‐of‐function mutation that represents the first loss‐of‐function Gα_11_ mutation to be located within the Gα‐subunit switch regions. The switch regions are comprised of three flexible peptide loops, which are highly conserved among all classes of Gα‐subunits, and undergo substantial conformational changes depending on the guanine nucleotide‐bound state of the Gα subunit.^30^ In the GTP‐bound state, these peptide loops switch their conformation to one that facilitates coupling with downstream effector proteins.^31^ The Phe220Ser mutation is located in the switch II region, which is predicted to bind and activate PLC,^11^ and our in vitro assessment of Ca^2+^_i_ and pERK responses in cells expressing the mutant Ser220 Gα_11_ demonstrated an impairment of signaling that is, at least in part, mediated by PLC.^26^, ^32^ Furthermore, our mutagenesis studies showed that Phe220 forms part of a group of hydrophobic residues that are required for coupling to PLC, and that Phe220 is located in a key region within this hydrophobic cleft, such that substitution of the hydrophobic Phe220 residue for a hydrophilic positively (Arg) or negatively (Glu) charged residue resulted, respectively, in a Gα_11_ gain‐of‐function or loss‐of‐function, thereby indicating that mutations of Phe220 can either retard or enhance the switch II conformational changes that occur upon G‐protein activation.

These studies have involved a comprehensive investigation of the effect of FHH2‐associated Gα_11_ mutations on PLC signaling at both the cytosolic (ie, Ca^2+^_i_ and pERK) and transcriptional (ie, NFAT and SRE reporter) level, and demonstrate that all readouts are affected by FHH2 mutations. The need to measure several signal outputs is increasingly important as recent studies have shown that GPCRs can couple to multiple G‐proteins to enhance their signaling capabilities and respond in diverse tissues.^33^, ^34^ Indeed, this is particularly the case in Gα_11_ mutant cells, where it has been hypothesized that other G‐proteins, in particular Gα_q_, may compensate for some of the functions of Gα_11_, giving rise to the milder phenotype observed in FHH2.^5^, ^6^ Our studies of the transcriptional events downstream of CaSR activation indicate that Ca^2+^_i_ signaling is more severely impaired than ERK signaling, as the Ser220 Gα_11_ mutant cells have more robust SRE signals than NFAT signals. MAPK signaling is known to occur downstream of several G‐proteins and can be used as a surrogate to assess multiple G‐protein families.^32^, ^35^ The differences observed in Ca^2+^_i_ signaling compared to ERK signaling in Gα_11_ Ser220 mutant cells indicates that Gα_11_ may preferentially signal via Ca^2+^_i_, or that other G‐proteins are able to compensate for a reduction in Gα_11_‐mediated ERK responses in FHH2 mutant cells.

The Gα_11_ protein is considered to be ubiquitously expressed,^36^ thus raising the possibility that germline Gα_11_ mutations may be associated with non‐calciotropic phenotypes. Indeed, in addition to constipation most of the affected family members had folliculitis or eczematous skin lesions, which may potentially represent a non‐calciotropic phenotype of FHH2. This is plausible as somatic activating Gα_11_ mutations have been reported to cause cutaneous pigmentation disorders such as dermal melanocytosis,^37^ and studies of atopic dermatitis, which is characterized by impaired epithelial barrier function and eczematous skin lesions, have revealed that G_q/11_‐coupled chemokine receptors facilitate the migration of inflammatory cells within the dermis.^38^ However, such roles of loss‐of‐function Gα_11_ mutations in cutaneous disorders remain to be elucidated, and these may be facilitated by studies of additional FHH2 patients and appropriate mouse models.

Our studies have also shown that cinacalcet‐mediated allosteric modulation of the CaSR successfully rectified the loss‐of‐function associated with the Phe220Ser Gα_11_ mutation in vitro and in vivo. However, a higher concentration of cinacalcet was required to normalize the signaling responses of cells expressing the Gα_11_ mutant Ser220, when compared to other FHH2‐causing Gα_11_ mutants.^10^ These in vitro findings suggest that loss‐of‐function mutations located within the Gα_11_ switch regions may alter the efficacy of calcimimetic compounds, and it is notable that the hypercalcemic proband with a Phe220Ser Gα_11_ mutation required an increase in the dose of cinacalcet from 30 to 60 mg daily to normalize the elevated serum calcium concentrations (Fig. 2 *C*). Moreover, cinacalcet in the patient exerted an inhibitory effect on PTH secretion, as observed by a normalization of serum PTH concentrations. Cinacalcet may be rectifying the impaired signaling by the mutant Phe220Ser Gα_11_ by increasing signaling through the WT Gα_11_, which is present endogenously in HEK293 cells and the cells of the heterozygous patient, and/or compensatory signaling by the closely‐related Gα_q_, which activates the same signaling pathways downstream of CaSR. Importantly, hypocalcemia or adverse effects such as nausea and vomiting, which may affect >25% of patients,^39^ were not observed in the patient, despite receiving a higher dose of cinacalcet.

In conclusion, we have identified a FHH2‐causing mutation, which affects the Gα_11_ switch region and disrupts PLC‐mediated signaling, and have also shown that cinacalcet can rectify these signaling disturbances and be used successfully to treat the hypercalcemia caused by this germline loss‐of‐function Gα_11_ mutation.

## Disclosures

FMH and RVT have received grant funding from NPS/Shire Pharmaceuticals and GlaxoSmithKline for unrelated studies involving the use of calcium‐sensing receptor allosteric inhibitors. RVT has also received grants from Novartis Pharma AG and the Marshall Smith Syndrome Foundation for unrelated studies. All other authors confirm no conflict of interest.

## Supporting information

Supporting Data S1.
