# Supplementary material for: Cinacalcet Rectifies Hypercalcemia in a Patient With Familial Hypocalciuric Hypercalcemia Type 2 (FHH2) Caused by a Germline Loss‐of‐Function Gα11 Mutation
Source: J Bone Miner Res. 2017 Sep 22;33(1):32–41. doi: 10.1002/jbmr.3241 (PMC5813271; doi:10.1002/jbmr.3241)
Supplement: Supplementary file 1 — Supporting Data S1. [file JBMR-33-32-s001.docx]

**SUPPLEMENTARY APPENDIX**

**Cinacalcet rectifies hypercalcemia in a patient with familial hypocalciuric hypercalcemia type 2 (FHH2) caused by a germline loss-of-function Gα_11_ mutation**

Caroline M. Gorvin^1^, Fadil M. Hannan^1,2^, Treena Cranston^3^, Helena Valta^4^, Outi Makitie^4^ , Camilla Schalin-Jantti^5,6^, Rajesh V. Thakker^1^

# Supplemental Figures and Tables

**Supplemental Figure 1 Structural characterization of the Phe220Ser of the Gα_11_ mutation**

**
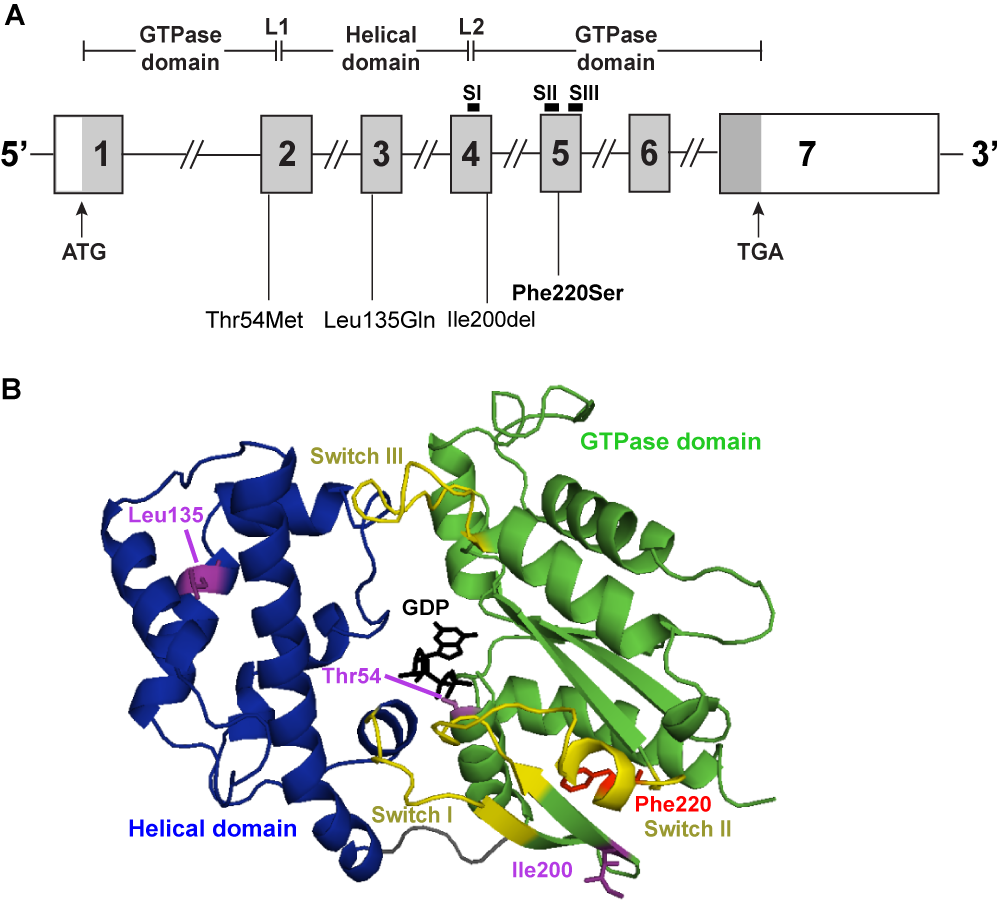
**

(**A**) Schematic representation of the genomic organisation of the human *GNA11* gene showing locations of FHH2-causing mutations^(^[^1^](#_ENREF_1)^,^[^2^](#_ENREF_2)^)^. The FHH2 mutation, Phe220Ser, described within this manuscript is shown in bold. The *GNA11* gene consists of 7 exons. Coding regions are shaded gray and untranslated regions are represented by open boxes. The GTPase domain (encoded by exon 1, 5’ portion of exon 2, 3’ portion of exon 4 and exons 5-7) is connected to the helical domain (encoded by the 3’ portion of exon 2, exon 3, and 5’ portion of exon 4) by the linker 1 (L1) and linker 2 (L2) peptides. Three flexible regions, termed switch regions I-III (SI-SIII), undergo conformational changes on Gα_11_ activation, and are encoded by exons 4 and 5. (**B**) Homology model of the Gα­_11_ protein comprising the Gα helical (blue) and GTPase (green) domains. GDP (black) is bound at the interdomain interface. Switches I-III are indicated in yellow. The 3 previously reported residues mutated in FHH2^(^[^1^](#_ENREF_1)^,^[^2^](#_ENREF_2)^)^ are shown in purple. The mutated Phe220 residue, identified by this study, is shown in red.

### Supplemental Figure 2 Expression of wild-type Gα_11_ and Gα_11_ mutants of residue 220 by transient transfection of appropriate constructs in HEK-CaSR cells

**
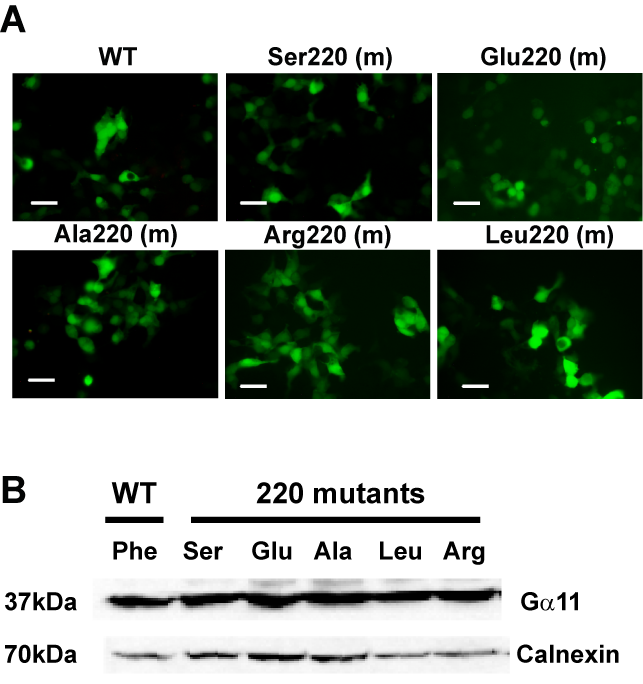
**

(**A**) Fluorescence microscopy of HEK293 cells stably expressing CaSR (HEK-CaSR) and transiently transfected with WT or mutant (m) residue 220 pBI-CMV2-*GNA11* constructs. GFP expression in these cells indicated successful transfection and expression by these constructs. Bar indicates 10μm. (**B**) Western blot analysis of lysates from HEK-CaSR cells transiently transfected with WT or mutant (m) pBI-CMV2-*GNA11* constructs. Transient transfection with WT or mutant expression constructs resulted in overexpression of Gα_11_ and GFP. Calnexin, a housekeeping protein, was used as a control.

### Supplemental Figure 3 Effect of mutating Phe220 Gα_11_ residue on Ca^2+^_i_ in HEK-CaSR cells

**
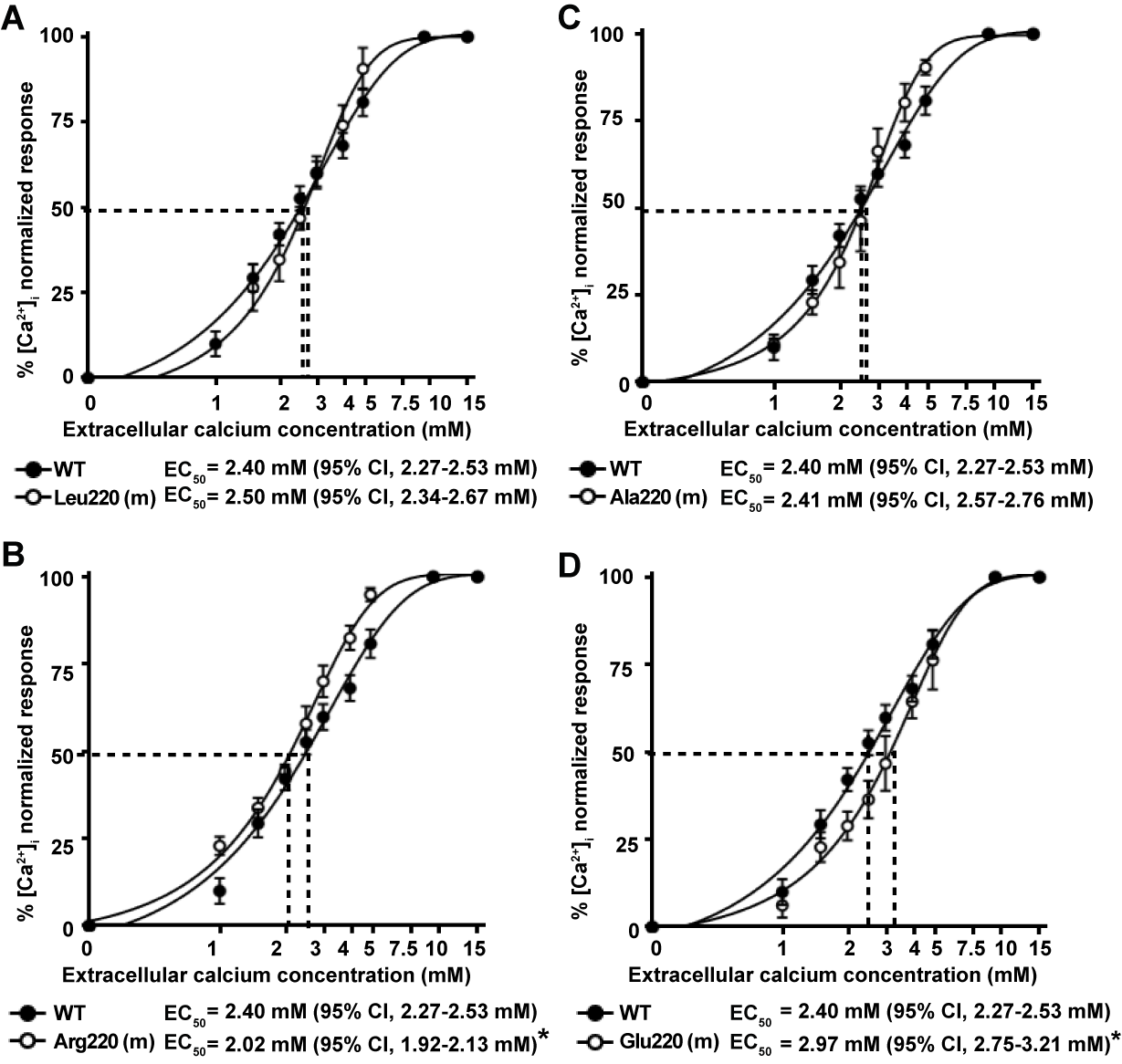
**

The Ca^2+^_i_ response to changes in [Ca^2+^]_e_ of cells expressing WT Gα_11_ or Gα_11_ mutants (**A**) Leu220, (**B**) Ala220, (**C**) Arg220, (**D**) Glu220 is shown as the mean±SEM of 6-12 transfections. The mean half-maximal values of each curve are shown underneath each graph. Statistical analyses were performed using the *F-*test. The Arg220 Gα_11_ mutant caused a leftward shift, and the Glu220 mutant caused a rightward shift in the concentration-response curve.

**Supplemental Figure 4** **Structural characterization of the Phe220Arg Gα_11_ mutation**

**
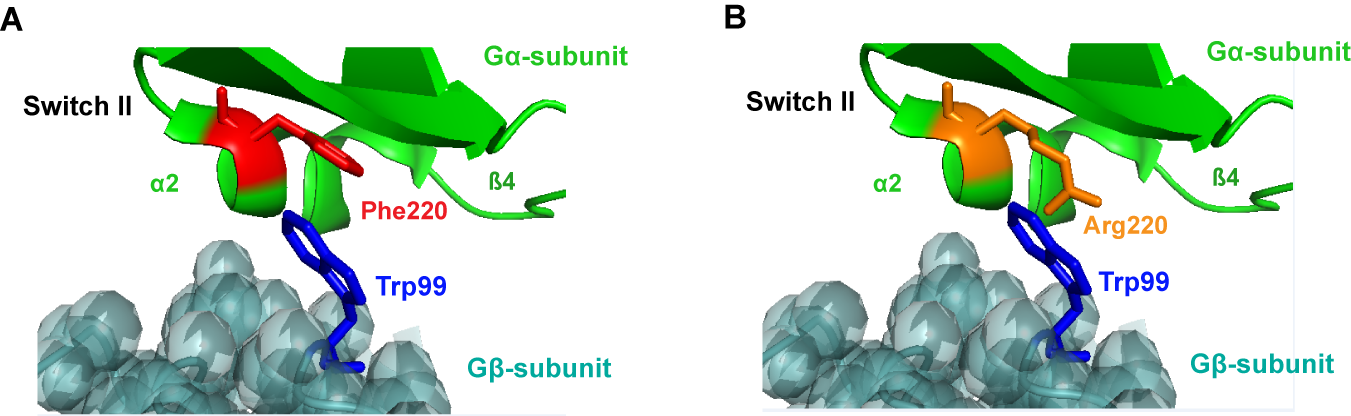
**

(**A**) Homology model of the region of the Gα­_11_ subunit (green) that interacts with the Gβ subunit (blue, space-filling). The Gα-βγ binding region is hydrophobic, and Phe220 (red) may contribute to this hydrophobicity, but is not predicted to be involved in Gβγ interaction. (**B)** Mutation to Arg220, a hydrophilic amino acid, brings the residue into close proximity with the Trp99 residue on the Gβ-subunit, and introduction of this charged residue is predicted to have a repulsive action on the Gα-βγ interaction region, leading to prolonged activation of the Gα-subunit.

###

### Supplemental Figure 5 Expression of Gα_11_ mutants of the switch II-α3 hydrophobic cluster by transient transfection of appropriate constructs in HEK-CaSR cells

**
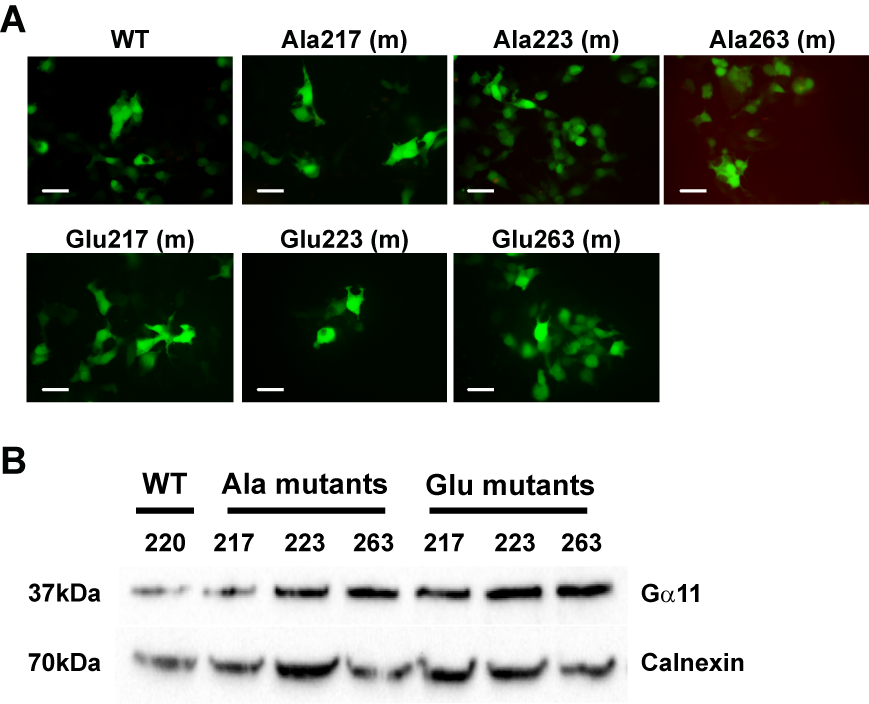
**

(**A**) Fluorescence microscopy of HEK293 cells stably expressing CaSR (HEK-CaSR) and transiently transfected with pBI-CMV2-*GNA11* constructs of WT, or alanine (Ala) and glutamic acid (Glu) Gα_11_ mutants (m) of hydrophobic residues at positions 217, 223 and 263. GFP expression in these cells indicated successful transfection and expression by these constructs. Bar indicates 10μm. (**B**) Western blot analysis of lysates from HEK-CaSR cells transiently transfected with WT or mutant (m) pBI-CMV2-*GNA11* constructs. Transient transfection with WT or mutant expression constructs resulted in overexpression of Gα_11_ and GFP. Calnexin, a housekeeping protein, was used as a control.

### Supplemental Figure 6 Effect on Ca^2+^_i_ signaling of mutation of residues within the switch 2-α3 hydrophobic cluster of Gα_11_ to alanine or glutamic acid residues

**
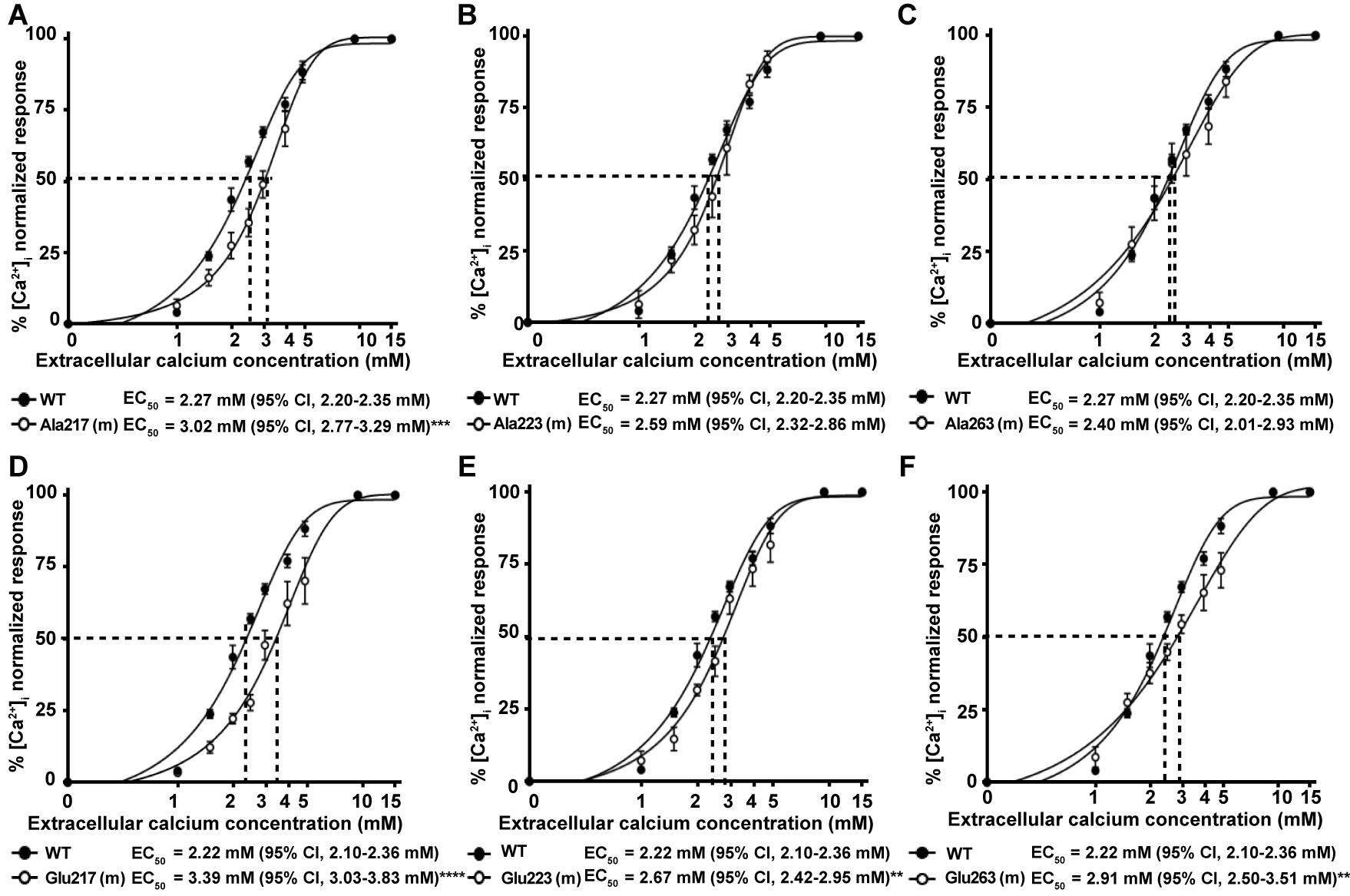
**

The Ca^2+^_i_ response to changes in [Ca^2+^]_e_ of cells expressing WT Gα_11_ or alanine (Ala) mutants (m) at Gα_11­_ residues (**A**) 217, (**B**) 223 or (**C**) 263. The Ala217 Gα_11_ mutant caused a rightward shift in the concentration-response curve. The Ca^2+^_i_ response to changes in [Ca^2+^]_e_ of cells expressing WT Gα_11_ or glutamic acid (Glu) mutants (m) at Gα_11_ residues (**A**) 217, (**B**) 223 or (**C**) 263. All 3 glutamic acid Gα_11_ mutants caused a rightward shift in the concentration-response curve**.** Data are shown as the mean±SEM of 4-8 transfections. The mean half-maximal values of each curve are shown underneath each graph. Statistical analyses were performed using the *F-*test.

### Supplemental Table 1 List of publically accessible databases of large-scale sequencing data examined for the presence of sequence variants

| **Database** | **Number of samples^*^** | **Website** | **Reference** |
| --- | --- | --- | --- |
| 1000 Genomes | 2,504 genomes | <http://browser.1000genomes.org> | ^(^[^3^](#_ENREF_3)^)^ |
| National Heart, Lung and Blood Institute (NHLBI) Exome Sequencing Project | 6,503 exomes | http://evs.gs.washington.edu/EVS/ | - |
| Exome Aggregation Consortium (ExAC) | 60,706 exomes | exac.broadinstitute.org | ^(^[^4^](#_ENREF_4)^)^ |

*number of samples on accession date of May 2017. -, no reference available

# References

1. Gorvin CM, Cranston T, Hannan FM, Rust N, Qureshi A, Nesbit MA, et al. G-Protein Subunit-alpha11 Loss-of-Function Mutation, Thr54Met, Causing Familial Hypocalciuric Hypercalcemia Type 2 (FHH2). Journal of bone and mineral research : the official journal of the American Society for Bone and Mineral Research. Jan 5 2016;31:1200-6.

2. Nesbit MA, Hannan FM, Howles SA, Babinsky VN, Head RA, Cranston T, et al. Mutations affecting G-protein subunit alpha11 in hypercalcemia and hypocalcemia. The New England journal of medicine. Jun 27 2013;368(26):2476-86.

3. Genomes Project C, Auton A, Brooks LD, Durbin RM, Garrison EP, Kang HM, et al. A global reference for human genetic variation. Nature. Oct 1 2015;526(7571):68-74.

4. Lek M, Karczewski KJ, Minikel EV, Samocha KE, Banks E, Fennell T, et al. Analysis of protein-coding genetic variation in 60,706 humans. Nature. Aug 18 2016;536(7616):285-91.
